# Exploring Community Attitudes Towards People Labelled as Institutional Child Sex Offenders

**DOI:** 10.5964/sotrap.14631

**Published:** 2024-07-31

**Authors:** Tiffany E. Taylor, Andy Williams

**Affiliations:** 1 School of Criminology and Criminal Justice, University of Portsmouth, Portsmouth, United Kingdom; University Medical Center Mainz, Mainz, Germany

**Keywords:** child sexual offending, institutional sexual abuse, community attitudes and perceptions, reintegration, rehabilitation

## Abstract

The sexual abuse of children perpetrated by persons who gain access to the child through their roles within child serving institutions, referred to here as institutional child sexual abuse, appears underexplored within the research community despite gaining considerable attention in the media. This study is a preliminary exploration of the stigmatization of individuals labelled as institutional child sexual offenders (ICSO). We recruited 347 community-based participants for an online survey regarding their desired social distance from, and attitudes towards, people labelled as ICSO as compared to those labelled as sexual offenders (SO). We utilized the CATSO, an 18-item attitudinal scale that measures attitudes towards people labelled as sex offenders, and the Bogardus social distance scale which measures the desired level of distance from outgroups. ICSO condition scores were higher than SO scores on the CATSO and lower than SO scores on the Bogardus. Scores for both scales indicate more negative attitudes and increased social desistance towards people labelled as ICSO than towards those labelled as SO. These preliminary findings support the identification of people labelled as ICSO as unique SO subgroup.

## Highlights

- The study examines community attitudes towards people labelled as institutional child sex offenders (ICSO).
- Attitudes towards people labelled as ICSO were more negative than attitudes towards people bearing the general label of sex offender (SO).
- Respondents desired greater social distance from people labelled as ICSO than from people labelled as SO.

Institutional child sexual abuse (ICSA) is a form of sexual abuse (SA) with unique characteristics that appears to be under-researched as a unique phenomenon (Hartley & Bartels, 2022; Socia et al., 2021). A number of those characteristics pertain to the person accused of committing SA (Falkenbach et al., 2019; McAlinden, 2018; Sullivan & Beech, 2004; Turner & Briken, 2015) differentiating people accused of committing ICSA from people accused of other forms of SA (Turner et al., 2014). There is a growing body of research on the negative impacts and stigmatizing effect that being labelled as a sex offender (SO) or perceived as a pedophile has on a person (Harris & Socia, 2016; Jahnke et al., 2015; Lowe & Willis, 2022) and it is important to differentiate between the person and the act. Research into community attitudes and public perceptions of people labelled as sex offenders typically does not differentiate between child sexual abuse (CSA) and SA (Hartley & Bartels, 2022; Seto et al., 2015). There is only limited research on attitudes towards people labelled as child sex offenders (CSO) (Hartley & Bartels, 2022) and no research on attitudes towards those labelled as institutional child sex offenders (ICSO) specifically has been found. Given the unique characteristics of ICSA and higher stigmatization of persons perceived as pedophiles, we anticipate that community member attitudes towards the ICSO label will differ from attitudes towards the SO label and thus the current study explores the impact of the ICSO label on community members’ attitudes.

## Institutional Child Sexual Offender Label

CSA is predominantly differentiated as intra- and extra- familial (Hartley & Bartels, 2022; Seto et al., 2015) but people labelled as ICSOs fall somewhere between as they are neither related to their victims nor strangers. They are the daycare providers, clergy members, teachers, youth group workers or coaches that parents entrust the care of their children to.

Several characteristics differentiate people labelled as ICSOs from people labelled as other extra-familial offenders (Falkenbach et al., 2019; McAlinden, 2018; Turner & Briken, 2015). Compared to people labelled as extra-familial CSOs, people labelled as ICSOs are considerably older with higher intelligence and greater educational attainment, less likely to have antisocial tendencies, but more likely to have atypical, particularly pedophilic, sexual interests (Falkenbach et al., 2019; Sullivan & Beech, 2004; Sullivan et al., 2011; Turner et al., 2014). People labelled as ICSOs also differ from people labelled as other extra-familial CSOs by the characteristics they share with people labelled as intra-familial CSOs. Like people labelled as intra-familial CSOs, people labelled as ICSOs frequently engage in pre-abuse grooming behaviours, manipulating the environment to facilitate SA (McAlinden, 2018; Sullivan & Beech, 2004; Sullivan et al., 2011; Turner & Briken, 2015). Due to their roles in youth serving organisations, people labelled as ICSOs have access to children and opportunities to offend that are similar to those of people labelled as intra-familial CSOs (McAlinden, 2018; Seto et al., 2015). It would appear that, due to not fully aligning with extra-familial characteristics and showing clear overlap with some intra-familial characteristics, the ICSO label may be a unique typology within the broader SO label (Turner & Briken, 2015; Turner et al., 2014).

## The Impact of Community Attitudes Towards People Labelled as Sexual Offenders

People labelled as SOs commonly face criminal justice system restrictions that are intended to reduce recidivism by restricting the movement and activities of released offenders (Hanson et al., 2018; Malinen et al., 2014). These policies are largely driven by community members’ attitudes towards people labelled as SOs (Malinen et al., 2014; Socia et al., 2021; Willis et al., 2013) making these attitudes an area of considerable importance. Community members’ attitudes to ex-offenders re-entering the community have consistently been found to be negative, making reintegration difficult. As community integration is a protective factor against re-offense (Willis et al., 2013) such negative attitudes can seriously impact an ex-offender’s successful rehabilitation.

The negative attitudes of community members towards people labelled as SOs in particular are typically highly punitive, favouring stiff sentences and only grudging acceptance of an accused offender’s release (Olver & Barlow, 2010). In the community people labelled as SOs struggle to find housing and meaningful employment and noncriminal community members treat them with suspicion and apprehension (Rydberg, 2018). Additionally, many people labelled as SOs face proximity restrictions for places children frequent (Rydberg, 2018), which can present employment barriers for people labelled as ICSOs given their previous employment, training, and experience is in fields such as education, sports, or religion that they may no longer be permitted to work in. However, research on SO recidivism finds most released offenders do not re-offend (Hanson et al., 2018) indicating that community attitudes are incommensurate with the level of risk and that person specific restrictions commensurate to an individual’s level of risk could be more appropriate.

## Present Study

The aim of the present study was to explore public opinion of individuals labelled as institutional child sexual offenders by surveying community members on their desired social distance from, and attitudes towards, people labelled as ICSOs. The study extended current community attitudes towards people labelled as SO research by narrowing the offender population to people labelled as ICSOs. The study explored whether there were differences in attitudes towards people labelled as SOs and people labelled as ICSOs. The specific hypotheses tested were:

1. Community members’ attitudes towards people labelled as sex offenders will be negative.
2. Community members’ attitudes will be more negative towards people labelled as institutional child sex offenders than towards people labelled as sex offenders.

## Method

### Participants

We recruited English speaking participants aged 18 or older (*M* = 34.44, *SD* = 12.02) from the global population to complete an online survey regarding attitudes towards people labelled as sexual offenders. 348 people responded to the survey invitation, one of whom was excluded for non-completion. Table 1 presents frequency statistics of participants’ demographic characteristics.

**Table 1:** Respondent Demographics Frequency Statistics

Variable	(*N* = 347)	
	*n*	%
Gender		
Male	148	42.65
Female	188	54.18
Other/Prefer not to say	11	3.17
Age		
18-21	59	17.00
22-29	103	29.68
30-39	87	25.07
40-49	53	15.27
> 50	45	12.97
Ethnicity		
Caucasian	237	68.30
Hispanic/Latino	48	13.83
Black/African/Caribbean	37	10.66
Mixed Race	16	4.61
Asian	6	1.73
North American Indian	2	0.58
Other/Prefer not to say	1	0.29
Education		
Secondary	76	21.90
College	54	15.56
Bachelors	138	39.77
Masters	67	19.31
Doctoral	8	2.31
Other/Prefer not to say	4	1.15
Employment		
Full-time	163	46.97
Part-time	34	9.80
Self-employed	35	10.09
Student	66	19.02
Unemployed	25	7.20
Other/Prefer not to say	24	6.92
Marital Status		
Married	136	39.19
Single	102	29.39
Relationship	87	25.07
Divorced/Widowed	13	3.75
Complicated	3	0.86
Other/Prefer not to say	6	1.73
Religion		
None	92	26.51
Christian	79	22.77
Catholic	55	15.85
Agnostic/Atheist	45	12.97
Spiritual	14	4.03
Other/Prefer not to say	62	17.87
How Religious		
Not	169	48.70
Moderately	73	21.04
Slightly	71	20.46
Very	20	5.76
Other/Prefer not to say	14	4.03
Sex Offender Experience		
None	133	38.33
Knows Victim	129	37.18
Knows Offender	10	2.89
Knows Victim and Offender	36	10.37
Field Knowledge	15	4.32
Field Knowledge and Knows Victim	14	0.04
Other/Prefer not to say	10	2.88
Parent		
Yes	177	51.01
No	164	47.26
Other/Prefer not to say	6	1.73
Continent		
Europe	180	51.87
North America	111	31.99
Africa	32	9.22
Australia	11	3.17
South America	9	2.59
Asia	3	0.86
Other/Prefer not to say	1	0.29

### Materials

#### Background Questions

Respondents were asked about their demographics and whether they had prior experience or familiarity with sexual abuse or persons accused of committing it.

#### Community Attitudes Towards Sex Offenders Scale (CATSO)

CATSO is an attitudinal scale, with high internal consistency, that measures community members perceptions, stereotypes, and attitudes towards people labelled as SOs (Church et al., 2008; Lowe & Willis, 2022; Willis et al., 2013). The CATSO consists of 18 questions grouped into four factors: *social isolation* (Questions 6, 7, 8, 14, 16); *capacity to change* (Questions 1, 2, 11, 12, 18); *severity/dangerousness* (Questions 4, 9, 13, 15, 17); and *deviancy* (Questions 3, 5, 10) (Church et al., 2008). Each question is scored on a six-point forced-choice Likert scale from strongly disagree (1) to strongly agree (6), three items are reverse-coded, with total scores ranging from 18 – 108, higher scores indicate more negative attitudes (Willis et al., 2013).

#### Bogardus Social Distance Scale

Introduced in the 1920s to measure racial acceptance, the Bogardus scale is used to understand intergroup social acceptance by measuring the social distance, or lack of proximity, community members desire from marginalized members of society (Malinen et al., 2014; Mather et al., 2017; Willis et al., 2013). The Bogardus scale is a ranked question with seven options ranging from accepting the outgroup member into the family through marriage (1) to excluding them from the country (7), with higher scores indicating increased social distance desired from the outgroup (Mather et al., 2017).

#### Study Definitions

The study defined people labelled as ICSOs as *an adult offender who had committed a sexual offense against a child whom they had access to through the course of their duties at a school, religious institution, sports organisation, or similar environment that the child attended* and sexual offenses against a child victim *as acts that may or may not involve direct physical contact with the child and may include acts such as sexual abuse (including rape), sexual interference, sexual exploitation, and invitation to sexual touching*.

### Procedure

Members of the global population were recruited through social media and Prolific. Recruitment ended after 2 weeks with 348 respondents so the first author could analyse the data for their master's thesis (see Taylor, 2023). The survey was hosted on JISC where respondents acknowledged the sensitive nature of the topic and consented to participate. Respondents answered 11 background questions followed by the CATSO and Bogardus scales. Then the study definitions were presented followed by the CATSO and Bogardus scales with the questions adapted to ask about people labelled as ICSOs. All participants received the questions in the same order to avoid the possibility of exposure to the ICSO condition inadvertently influencing responses to the SO condition if participants had the ICSO condition first.

### Data Analysis

Bivariate comparisons were conducted for differences in attitudes towards people labelled as SO and people labelled as ICSO independent *t*-tests were conducted for comparison of attitudes.

## Results

### Hypothesis 1: Community Members’ Attitudes Towards People Labelled as Sex Offenders Will Be Negative

Summary statistics for the CATSO are presented in Table 2. SO condition total scores, *M* = 54.49 (*SD* = 9.80), correspond to an item mean of *M* = 3.03 (*SD* = 0.54) indicating participants “probably disagreed” with the CATSO statements. Results for the Bogardus are presented in Table 3. SO condition total score, *M* = 16.82 (*SD* = 7.57) fell well below the 27.5 midpoint indicating responses were in the “most definitely not” category.

**Table 2:** CATSO Results for SO and ICSO Conditions

CATSO Questions	Full Sample (*N* = 347)			
	SO		ICSO	
	*M*	*SD*	*M*	*SD*
1. With support and therapy, someone who committed a [sexual] offense can learn to change their behaviour (R)	3.44	1.45	4.03	1.57
2. People who commit [sex] offences should lose their civil rights (e.g., voting and privacy)	3.41	1.84	3.70	1.90
3. People who commit [sex] offences want to have sex more often than the average person	2.70	1.39	2.73	1.30
4. Male sex offenders should be punished more severely than female sex offenders	1.60	1.13	4.07	1.78
5. Sexual fondling (inappropriate unwarranted touch) is not as bad as rape	2.43	1.58	2.25	1.52
6. [Sex] offenders prefer to stay home alone rather than be around lots of people	3.00	1.17	2.95	1.29
7. Most [sex] offenders do not have close friends	2.56	1.32	2.96	1.35
8. [Sex] offenders have difficulty making friends even if they try real hard	2.84	1.29	2.88	1.35
9. The prison sentences [sex] offenders receive are much too long when compared with the sentence lengths for other crimes (R)	5.22	1.03	5.24	1.15
10. [Sex] offenders have high rates of sexual activity	3.12	1.27	2.88	1.23
11. Trying to rehabilitate a [sex] offender is a waste of time	3.21	1.62	3.66	1.73
12. [Sex] offenders should wear tracking devices so their location can be pinpointed at all times	3.92	1.70	4.33	1.80
13. Only a few [sex] offenders are dangerous (R)	5.12	1.15	5.48	0.90
14. Most [sex] offenders are unmarried men	2.39	1.24	2.56	1.29
15. Someone who uses emotional control when committing a sex offense is not as bad as someone who uses physical control when committing a sex offense	1.72	1.22	1.72	1.13
16. Most [sex] offenders keep to themselves	3.39	1.47	3.11	1.45
17. A sex offense committed against someone the perpetrator knows is less serious than a sex offense committed against a stranger	1.35	0.87	2.59	1.64
18. Convicted [sex] offenders should never be released from prison	3.21	1.67	3.80	1.86
Total (18 items)	54.49	9.80	60.76	11.93

*Note.* Scored 1 (strongly disagree) to 6 (strongly agree), no neutral. (R) denotes reverse coded variable. ICSO adaptations: Q4 “Institutional child sex” replaced "Male sex" and “other” replaced "female". Q17 "an adult" replaced “someone the perpetrator knows” and "child" replaced “stranger”. Q5 and 15 were not changed. In all other questions “Institutional child sex” replaced [sex].

**Table 3:** Social Distance Scale (SDS) Results for SO and ICSO Conditions

Variable	*(N* = 347)			
	SO		ICSO	
	*M*	*SD*	*M*	*SD*
Neighbour	1.62	0.93	1.44	0.80
Colleague	1.66	0.93	1.41	0.80
Boss	1.47	0.84	1.30	0.70
Acquaintance	1.64	0.92	1.36	0.74
Group Member	1.80	1.07	1.39	0.79
Friend	1.43	0.89	1.22	0.64
Spouse	1.31	0.76	1.18	0.60
Son in law	1.38	0.80	1.25	0.65
Employee	1.59	0.96	1.46	0.86
Tenant	1.67	1.00	1.51	0.89
Introduce to people	1.28	0.68	1.21	0.62
Total	16.82	7.57	14.72	6.30

*Note.* Scored 1 (most definitely not) to 5 (most definitely), 3 is neutral.

### Hypothesis 2: Community Members’ Attitudes Will Be More Negative Towards People Labelled as ICSO Than Towards People Labelled as SO

Paired-samples *t*-tests were conducted on both the CATSO and Bogardus scores to determine whether respondents had more negative attitudes toward people labelled as ICSOs than they did to people labelled as SOs. Significant difference in attitudes were found on the CATSO (SO (*M* = 54.49, *SD* = 9.80), ICSO (*M* = 60.76, *SD* = 11.93), *t*(346) = -14.22, *p* < .001, *d* = 0.57, medium effect) and Bogardus (SO (*M* = 16.80; *SD* = 7.55), ICSO (*M* = 14.73; *SD* = 6.26), *t*(346) = 8.89, *p* < .001, *d* = 0.30, small to medium effect). CATSO factors were analysed separately and significant differences in attitudes were found for the *severity/dangerousness* (SO (*M* = 15.01, *SD* = 2.56), ICSO (*M* = 17.58, *SD* = 2.99), *t*(346) = - 14.21, *p* < .001, *d =* 0.92, large effect), *capacity to change* (SO (*M* = 17.14, *SD* = 6.48), ICSO (*M* = 19.46, *SD* = 6.96), *t*(346) = -10.43, *p* < .001, *d* = .035, small effect), and *deviancy* (SO (*M* = 8.20, *SD* = 2.81), ICSO (*M* = 7.85, *SD* = 2.79), *t*(346) = 3.90, *p* < .001, *d* = 0.12, negligible effect) factors.

## Discussion

The study explored community member attitudes differed if a person was labelled as an SO or ICSO. First, we measured attitudes towards people labelled as SOs to determine whether community members’ attitudes towards people labelled as SOs were negative then examined whether attitudes were more negative towards people labelled as ICSOs than people labelled as SOs. As hypothesized, attitudes towards people labelled as SO were negative, and attitudes towards people labelled as ICSO were more negative. CSA is seen as one of the worst forms of criminal violence (Hartley & Bartels, 2022; Lowe & Willis, 2022) and the people accused of committing it are commonly considered as the scourge of society (Malinen et al., 2014; Socia et al., 2021). ICSA is a form of CSA that gains considerable media attention when reported, and the people accused of committing it have violated the trust parents place in them to care for their children (Falkenbach et al., 2019; McAlinden, 2018). As such, it is not surprising that community members view ICSA even more negatively than CSA.

Hypothesis 1 was partially supported as Bogardus scores showed respondents “most definitely [did] not” want people labelled sex offenders in their communities while CATSO were less definitive as respondents said they “probably disagreed” with the CATSO statements. Combined, results indicate people labelled as SOs are unwanted in the community and community members may disagree with cognitive beliefs pertaining to people labelled as SOs. Hypothesis 2 was fully supported as attitudes towards people labelled as ICSOs were more negative than attitudes towards people labelled as SOs. Similar to the people labelled as SO results, Bogardus scores indicated respondents “most definitely [did] not” want people labelled as either SO or ICSO in their communities. However, ICSO scores were lower than SO scores indicating an increased desire for social distance from people labelled as ICSOs. CATSO scores were in the “probably disagree” category but respondent scores were higher for people labelled as ICSOs than people labelled as SOs indicating less disagreement with the CATSO statements towards people labelled as ICSO. Looking at the CATSO factors, scores were a half-point higher for people labelled as ICSOs approaching “probably agree” on the *severity/dangerousness* and *capacity to change* factors indicating community members may consider people labelled ICSOs a greater risk than other people labelled as sex offenders.

The findings are consistent with existing research on attitudes towards, and desired social distance from, people labelled as sex offenders. Overall CATSO scores obtained were within one-point of prior studies (Malinen et al., 2014; Willis et al., 2013) and consistent with research measuring attitudes through means such as perceived dangerousness (Hartley & Bartels, 2022) and beliefs about punitive measures (Olver & Barlow, 2010; Socia et al., 2021). The average Bogardus scores below two indicating increased desire for social distance from people labelled as SOs are consistent with Willis et al. (2013) findings and with findings measuring social distance through other means (Malinen et al., 2014; Olver & Barlow, 2010). Findings that community members’ attitudes towards people labelled as ICSOs were more negative than attitudes towards people labelled as SOs is consistent with research that has found attitudes are more negative towards specifically labelled sex offenders (Harris & Socia, 2016; Lowe & Willis, 2022) and those with child victims (Socia et al., 2021) or perceived pedophilia (Jahnke et al., 2015) further illustrating that not all SO labels are seen equally and that attitudinal research into more specific subgroups needs to be conducted.

Labels are highly stigmatizing causing increased negative attitudes towards people who bear certain labels (Harris & Socia, 2016; Lowe & Willis, 2022) and people with perceived pedophilia are more stigmatized than people with other labels (Jahnke et al., 2015). The fear of being associated with stigmatized people can lead to the rejection of the individual by community members, employers, or landlords (Lowe & Willis, 2022; Rydberg, 2018). The negative attitudes towards people labelled as ICSOs reported in the study could suggest that a fear of being stigmatized could contribute to attempts to prevent disclosure or prevent potential ICSOs from seeking help to prevent offending. If so, the stigmatization and community rejection of people labelled as institutional child sex offenders may have an unintended effect of increasing the risk of (re)offence by restricting prosocial or treatment opportunities for people labelled as ICSOs.

Finally, the current study contributes to research on sex offender typologies supporting the identification of people labelled as ICSOs as a unique offender typology as the study found community members have different attitudes towards people labelled ICSOs than they do towards people labelled as SOs.

### Limitations and Future Work

The current study is not without limitations. To begin with, the study took a global approach drawing 347 respondents from all continents except Antarctica resulting in small sample sizes for individual countries and continents which limits generalizability of the findings. Another key limitation of the study was the test/re-test approach, responding to the same questions for both offender labels may have primed respondents and introduced an order-effect bias or respondent fatigue. The most significant limitation is that the current study compared people labelled as SOs to people labelled as ICSOs and did not compare people labelled as CSOs to people labelled as ICSOs. It may be that the significant difference in attitudes is due to the introduction of a child victim and that a comparison to people labelled as CSOs may have produced different results. Another potential limitation is the scales chosen for the study. There are a number of methods of measuring community attitudes and social acceptance and different measures may yield different results. Despite these limitations, the current study is a promising first foray into community members’ attitudes towards people labelled ICSOs and provides promising findings and directions for future research. Future research should compare attitudes towards people labelled as CSOs and people labelled as ICSOs using a between-groups approach with a large sample from a geographically defined region. Alternate scales and scoring methods, such as Mather et al.’s (2017) iScore for the Bogardus, should also be employed.

### Conclusion

Research into the attitudes of community members towards people labelled as sex offenders has focused predominantly on the broad sex offender label with limited research exploring attitudes towards more narrowly defined SO subgroups. The current study’s findings join works that show not all people labelled as sex offenders are seen equally and that attitudinal research needs to be conducted that considers offender subgroups such as work exploring people labelled as juvenile sex offenders (Harris & Socia, 2016) and people labelled as child sex offenders (Hartley & Bartels, 2022). Due to the non-homogeneity of people labelled as SOs and the weight community attitudes towards people labelled as SOs have on criminal justice system policies and the ability of people bearing SO labels to reintegrate into the community (Malinen et al., 2014; Socia et al., 2021; Willis et al., 2013), it is important to develop an understanding of community attitudes towards SO subgroups. More accurate perceptions of community member attitudes could be beneficial when determining policies and reintegration plans to aid in reducing recidivism and giving people bearing SO labels opportunities to be seen as a person and not what they are accused of having done. The fact that people labelled as ICSOs are some of the more sensationalised sex offenders reported in the media, and that reports of institutional child sexual abuse are on the rise, makes this specific subgroup a timely one to focus on.

## Data Availability

The original data cannot be provided due to the statement made to participants in the informed consent form that the data would not be shared.

## Related Versions

This data was collected as part of the requirements for the first author’s master’s thesis. The full thesis explores five hypotheses and can be found in Taylor (2023).

## References

[r1] Church, W. T., Wakeman, E. E., Miller, S. L., Clements, C. B., & Sun, F. (2008). The Community Attitudes Toward Sex Offenders scale: The development of a psychometric assessment instrument. Research on Social Work Practice, 18(3), 251–259. 10.1177/1049731507310193

[r2] Falkenbach, D. M., Foehse, A., Jeglic, E., Calkins, C., & Raymaekers, L. (2019). Sexual abuse within employment settings: A comparison of work-related, intra- and extra-familial child molesters. Sexual Abuse, 31(5), 524–542. 10.1177/1079063217708202 28643546

[r3] Hanson, R. K., Harris, A. J., Letourneau, E., Helmus, L. M., & Thornton, D. (2018). Reductions in risk based on time offense-free in the community: Once a sexual offender, not always a sexual offender. Psychology, Public Policy, and Law, 24(1), 48–63. 10.1037/law0000135

[r4] Harris, A. J., & Socia, K. M. (2016). What’s in a name? Evaluating the effects of the “sex offender” label on public opinions and beliefs. Sexual Abuse, 28(7), 660–678. 10.1177/1079063214564391 25542837

[r5] Hartley, M., & Bartels, R. M. (2022). Public perception of men who have committed intrafamilial and extrafamilial sexual offences against children. Sexual Abuse, 34(8), 1003–1028. 10.1177/10790632211062188 35259025 PMC9643821

[r6] Jahnke, S., Imhoff, R., & Hoyer, J. (2015). Stigmatization of people with pedophilia: Two comparative surveys. Archives of Sexual Behavior, 44(1), 21–34. 10.1007/s10508-014-0312-4 24948422

[r7] Lowe, G. T., & Willis, G. M. (2022). Do popular attitudinal scales perpetuate negative attitudes towards persons who have sexually offended? Journal of Sexual Aggression, 28(2), 231–243. 10.1080/13552600.2021.2009050

[r8] Malinen, S., Willis, G. M., & Johnston, L. (2014). Might informative media reporting of sexual offending influence community members’ attitudes towards sex offenders? Psychology, Crime & Law, 20(6), 535–552. 10.1080/1068316X.2013.793770

[r9] Mather, D. M., Jones, S. W., & Moats, S. (2017). Improving upon Bogardus: Creating a more sensitive and dynamic social distance scale. Survey Practice, 10(4). 10.29115/SP-2017-0026

[r10] McAlinden, A.-M. (2018). Organisational sex offenders and 'Institutional Grooming': Lessons from the Savile and other inquiries. In M. Erooga (Ed.), *Protecting children and adults from abuse after Savile: What organizations and institutions need to do* (pp. 72-99). Jessica Kingsley. https://pure.qub.ac.uk/files/140510907/3_Savile_Chapter_McAlinden_Final.pdf

[r11] Olver, M. E., & Barlow, A. A. (2010). Public attitudes toward sex offenders and their relationship to personality traits and demographic characteristics. Behavioral Sciences & the Law, 28(6), 832–849. 10.1002/bsl.959 20857417

[r12] Rydberg, J. (2018). Employment and housing challenges experienced by sex offenders during reentry on parole. Corrections: Policy, Practice and Research, 3(1), 15–37. 10.1080/23774657.2017.1369373

[r13] Seto, M. C., Babchishin, K. M., Pullman, L. E., & McPhail, I. V. (2015). The puzzle of intrafamilial child sexual abuse: A meta-analysis comparing intrafamilial and extrafamilial offenders with child victims. Clinical Psychology Review, 39, 42–57. 10.1016/j.cpr.2015.04.001 25935749

[r14] Socia, K. M., Rydberg, J., & Dum, C. P. (2021). Punitive attitudes toward individuals convicted of sex offences: A vignette study. Justice Quarterly, 38(6), 1262–1289. 10.1080/07418825.2019.1683218

[r15] Sullivan, J., & Beech, A. (2004). A comparative study of demographic data relating to intra- and extra-familial child sexual abusers and professional perpetrators. Journal of Sexual Aggression, 10(1), 39–50. 10.1080/13552600410001667788

[r16] Sullivan, J., Beech, A. R., Craig, L. A., & Gannon, T. A. (2011). Comparing intra-familial and extra-familial child sexual abusers with professionals who have sexually abused children with whom they work. International Journal of Offender Therapy and Comparative Criminology, 55(1), 56–74. 10.1177/0306624X09359194 20150651

[r17] Taylor, T. (2023). *Are they viewed differently? An exploration of community member attitudes towards institutional child sex offenders* [Unpublished master’s thesis]. University of Portsmouth.

[r18] Turner, D., & Briken, P. (2015). Child sexual abusers working with children – Characteristics and risk factors. Sexual Offender Treatment, 10(1), 1–12.

[r19] Turner, D., Rettenberger, M., Lohmann, L., Eher, R., & Briken, P. (2014). Pedophilic sexual interests and psychopathy in child sexual abusers working with children. Child Abuse & Neglect, 38(2), 326–335. 10.1016/j.chiabu.2013.07.019 24008098

[r20] Willis, G. M., Malinen, S., & Johnston, L. (2013). Demographic differences in public attitudes towards sex offenders. Psychiatry, Psychology and Law, 20(2), 230–247. 10.1080/13218719.2012.658206

